# p*K*_a_ of ubiquinone, menaquinone, phylloquinone, plastoquinone, and rhodoquinone in aqueous solution

**DOI:** 10.1007/s11120-017-0382-y

**Published:** 2017-04-12

**Authors:** Ryo Hasegawa, Keisuke Saito, Tomohiro Takaoka, Hiroshi Ishikita

**Affiliations:** 1 Department of Applied Chemistry, The University of Tokyo, 7-3-1 Hongo, Bunkyo-ku, Tokyo, 113-8654 Japan; 2 Research Center for Advanced Science and Technology, The University of Tokyo, 4-6-1 Komaba, Meguro-ku, Tokyo, 153-8904 Japan

**Keywords:** Photosystem II, Bacterial photosynthetic reaction centers, *Rhodobacter sphaeroides*, *Blastochloris viridis*, Green non-sulfur bacteria, Evolutionary transition

## Abstract

Quinones can accept two electrons and two protons, and are involved in electron transfer and proton transfer reactions in photosynthetic reaction centers. To date, the p*K*_a_ of these quinones in aqueous solution have not been reported. We calculated the p*K*_a_ of the initial protonation (Q^·−^ to QH^·^) and the second protonation (QH^−^ to QH_2_) of 1,4-quinones using a quantum chemical approach. The calculated energy differences of the protonation reactions Q^·−^ to QH^·^ and QH^−^ to QH_2_ in the aqueous phase for nine 1,4-quinones were highly correlated with the experimentally measured p*K*_a_(Q^·−^/QH^·^) and p*K*_a_(QH^−^/QH_2_), respectively. In the present study, we report the p*K*_a_(Q^·−^/QH^·^) and p*K*_a_(QH^−^/QH_2_) of ubiquinone, menaquinone, phylloquinone, plastoquinone, and rhodoquinone in aqueous solution.

**Electronic supplementary material:**

The online version of this article (doi:10.1007/s11120-017-0382-y) contains supplementary material, which is available to authorized users.

## Introduction

**Fig. 1:**
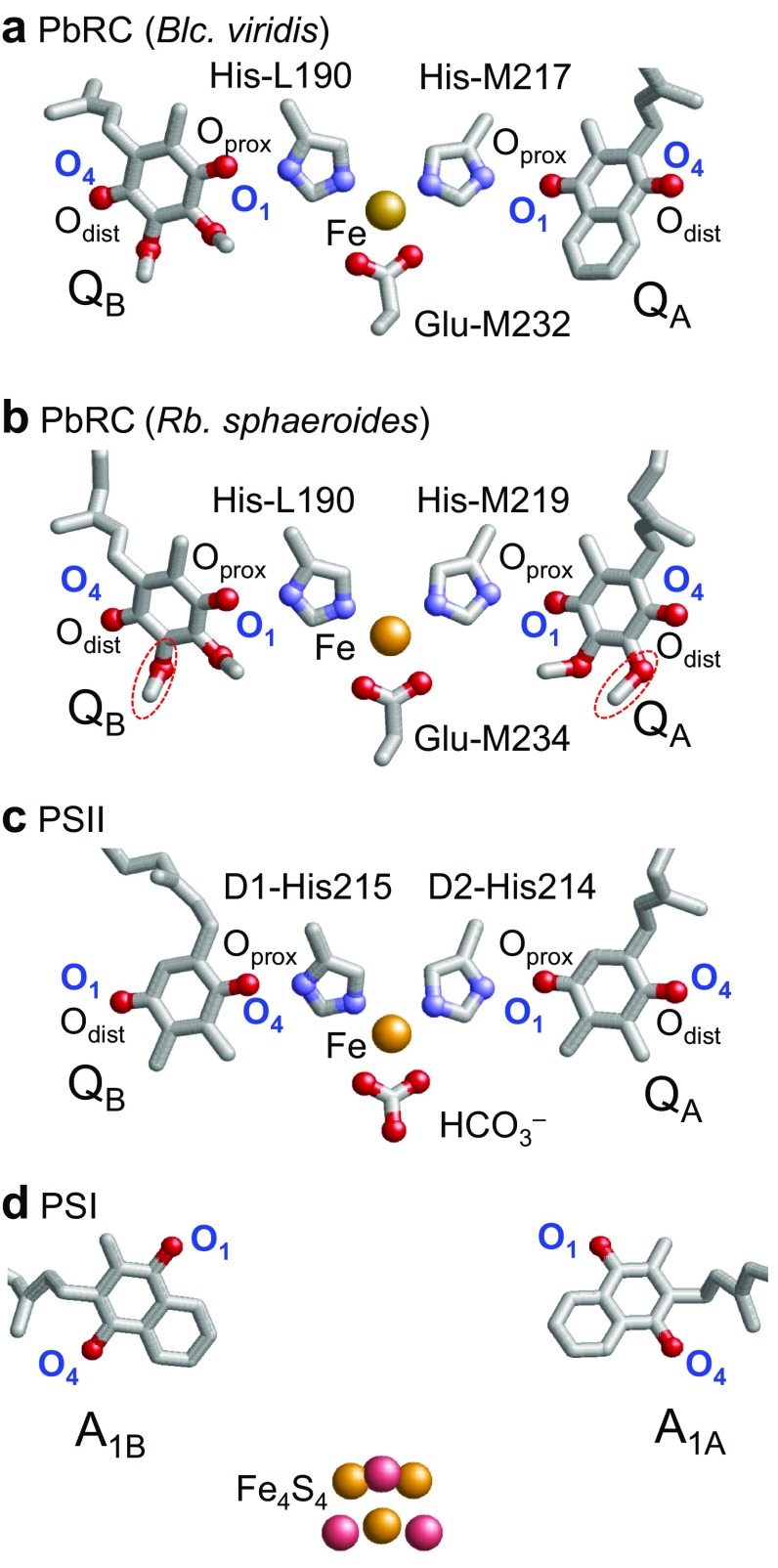
Quinones in photosynthetic reaction centers: **a** menaquinone as Q_A_ and ubiquinone as Q_B_ in bacterial photosynthetic reaction centers from *Blastochloris viridis* (*Blc. viridis*, PDB ID: 2I5N) (Li et al. 2006); **b** ubiquinone as Q_A_ and Q_B_ in bacterial photosynthetic reaction centers from *Rhodobacter sphaeroides* (*Rb. sphaeroides*, PDB ID: 3I4D), where the 2-methoxy group of each ubiquinone is *red circled*; **c** plastoquinone as Q_A_ and Q_B_ in PSII (PDB ID: 3ARC) (Umena et al. 2011); and **d** phylloquinone as A_1A_ and A_1B_ in PSI (PDB ID:1JB0) (Jordan et al. 2001). *Red* and *blue balls* indicate O and N atoms, respectively. In PbRC and PSII, O_prox_ and O_dist_ stand for O atoms of the quinones at the proximal and distal positions with respect to the non-heme Fe complex, respectively. Note that except for Q_B_ in PSII, O_prox_ is O1 and O_dist_ is O4 in PbRC and PSII

**Fig. 2:**
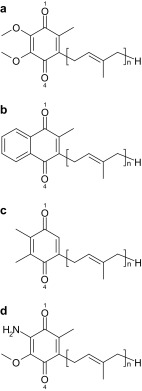
Molecular structures of **a** ubiquinone (*n* = 10), **b** menaquinone/phylloquinone (*n* = 3–9), **c** plastoquinone (n = 6–9), and **d** rhodoquinone (*n* = 10), where *n* is the number of isoprene units

Quinones can accept two electrons and two protons via the initial protonation of semiquinone, Q^·−^ to QH^·^, and second protonation of hydroquinone, QH^−^ to QH_2_. Ubiquinone serves as an electron acceptor at the Q_A_ and Q_B_ binding sites in reaction centers of purple bacteria (PbRC) from *Rhodobacter sphaeroides* (Fig. 1). Similarly, menaquinone (vitamin K_2_) is the acceptor at the Q_A_ site in PbRC from *Blastochloris viridis*, while phylloquinone (vitamin K_1_) is the active center at the A_1A_ and A_1B_ sites in photosystem I (PSI). In reaction centers of green non-sulfur bacteria from *Chloroflexus aurantiacus*, menaquinones are also located at both the Q_A_ and Q_B_ sites (Hale et al. 1983). It should be noted that phylloquinone and menaquinone have the same head-group structure (Fig. 2). Plastoquinone serves as an electron acceptor at the Q_A_ and Q_B_ sites in photosystem II (PSII) (Fig. 1) (Robinson and Crofts 1984; Rutherford et al. 1984; Okamura et al. 2000; Brettel and Leibl 2001; Wraight 2004). Rhodoquinone is a required cofactor for anaerobic respiration in *Rhodospirillum rubrum* (Okayama et al. 1968). Because rhodoquinone is assumed to have a higher p*K*_a_(Q^·−^/QH^·^) than ubiquinone, rhodoquinone-substituted PbRC has been used to investigate the mechanism of proton transfer to Q_B_ (e.g., Graige et al. 1999; Maroti et al. 2015).

In PbRC and PSII, Q_A_/Q_A_^·−^ acts as a one-electron redox couple and donates an electron to the second quinone Q_B_, without undergoing protonation itself. In contrast, Q_B_ reduction involves two consecutive one-electron reduction reactions with a series of associated proton uptake reactions (reviewed in Diner and Rappaport 2002; Renger and Renger 2008; Holzwarth 2008; Cardona et al. 2012; Muh et al. 2012; Petrouleas and Crofts 2005). Both Q_A_ and Q_B_ are located near the non-heme Fe^2+^ and the ligands to the Fe^2+^ (i.e., His-L190 and His-M217 (or M219) in PbRC and D1-His215 and D2-His214 in PSII) donate an H-bond to the carbonyl O atoms of quinones that are nearer to the Fe complex (O_prox_) (Fig. 1a–c). The carbonyl O atoms of quinones at the distal position (O_dist_) also form H-bonds with the proteins. On the other hand, in PSI, the non-heme Fe^2+^ is absent, but the Fe_4_S_4_ cluster F_X_ is located near the two A_1_ binding sites (Fig. 1d). For many 1,4-quinones, experimentally measured p*K*_a_(Q^·−^/QH^·^) in aqueous solution were summarized by Swallow (1982) and experimentally measured p*K*_a_(QH^−^/QH_2_) in aqueous solution were reported by Bishop and Tong (1965).

As far as we are aware, experimentally measured p*K*_a_(Q^·−^/QH^·^) and p*K*_a_(QH^−^/QH_2_) for ubiquinone, menaquinone, plastoquinone, and rhodoquinone in aqueous solution have not been reported, because of their insoluble hydrophobic isoprene side-chains (Fig. 2). The p*K*_a_(Q^·−^/QH^·^) for ubiquinone was roughly estimated to be 4.9 in aqueous solution (Swallow 1982) or measured to be 6.5 in methanol (Land and Swallow 1970; Swallow 1982). In theoretical studies by Cape et al. (2006), p*K*_a_(Q^·−^/QH^·^) for ubiquinone, plastoquinone, and rhodoquinone were calculated to be 5.35, 4.86, and 5.09, respectively (Cape et al. 2006), although p*K*_a_(Q^·−^/QH^·^) of rhodoquinone is assumed to be higher than p*K*_a_(Q^·−^/QH^·^) of ubiquinone (Graige et al. 1999). The p*K*_a_(QH^−^/QH_2_) for ubiquinone were measured to be 13.3 in 80% ethanol (Morrison et al. 1982). On the other hand, to calculate p*K*_a_(QH^−^/QH_2_) of Q_B_ in PbRC from *R. sphaeroides*, Zhu and Gunner used a p*K*_a_(QH^−^/QH_2_) of 10.7 for ubiquinone in aqueous solution in their theoretical studies (Zhu and Gunner 2005). Reliable p*K*_a_(Q^·−^/QH^·^) and p*K*_a_(QH^−^/QH_2_) values for ubiquinone, menaquinone, and plastoquinone in aqueous solution are required for understanding the mechanisms of reactions involving quinones in PbRC, PSI, and PSII. Here, we report the p*K*_a_(Q^·−^/QH^·^) and p*K*_a_(QH^−^/QH_2_) for ubiquinone, menaquinone, phylloquinone, and plastoquinone in aqueous solution, obtained by adopting a quantum chemical approach.

## Computational procedures

In the deprotonation reaction of the protonated state (AH) to deprotonated state (A^−^) in aqueous solution, p*K*_a_ is defined as

1 $$p{K_{\text{a}}}=\frac{{\Delta {G_{{\text{aq}}}}}}{{2.303RT}},$$

where Δ*G*_aq_ is the free energy difference between (AH) and (A^−^ + H^+^) (i.e., Δ*G*_aq_ = *G*_aq_(A^−^) − *G*_aq_(AH) + *G*_aq_(H^+^)), *R* is the gas constant, and *T* is the temperature. Δ*G*_aq_ can also be approximated as

2 $$\Delta {G_{{\text{aq}}}}={\text{ }}k\Delta {E_{{\text{QM}}/{\text{PCM}}}}+C,$$

where *k* is the scaling factor, Δ*E*_QM/PCM_ is the energy difference between AH and A^−^ in aqueous phase (i.e., Δ*E*_QM/PCM_ = *E*_QM/PCM_(A^−^) − *E*_QM/PCM_(AH)), which can be calculated using a quantum chemical (QM) approach with the polarizable continuum model (PCM) method, and *C* is the constant [“simple p*K*_a_ estimation with energy of the optimized geometry scheme” (Matsui et al. 2012)]. If p*K*_a_ of molecules are obtained at the same temperature, Eq. 1 can be written into Eq. 3 using Eq. 2,

3 $$p{K_{\text{a}}}=k'\Delta {E_{{\text{QM}}/{\text{PCM}}}}+C',$$

where *k*′ is the scaling factor and *C*′ is constant. To determine *k*′ and *C*′, we calculated Δ*E*_QM/PCM_ for nine 1,4-quinones whose experimentally measured p*K*_a_(Q^·−^/QH^·^) (Swallow 1982) and p*K*_a_(QH^−^/QH_2_) (Bishop and Tong 1965) are reported. We employed the unrestricted DFT method with the B3LYP functional and 6-31g++** basis sets, using the Gaussian (Frisch et al. 2004) program code with the PCM method, i.e., water molecules were considered implicitly. This treatment is more appropriate to describe H-bonds between quinones and bulk water molecules, in which the H-bond patterns are not unique. We evaluated all possible conformations of each protonated quinone (i.e., QH^·^ and QH_2_) regarding the –OH group orientation, and we took the energetically lowest conformation as the relevant structure. In the resulting structures, the –OH group was essentially in the plane of the quinone ring (SI Dataset 1 for atomic coordinates). Because the isoprene units are not composed of conjugated double bonds, the isoprene side-chain length *n* (Fig. 2) was set to 1 for the calculations of ubiquinone, menaquinone, phylloquinone, and plastoquinone. This could also reduce the number of the possible conformations. In fact, the length of the ubiquinone does not practically affect the energetics of ubiquinones, as demonstrated by the experimentally measured redox potential values of ubiquinone-1 and -10 in dimethylformamide being −611 and −602 mV versus saturated calomel reference electrode, respectively, essentially no difference (Prince et al. 1983).

## Results and discussion

### Correlation of calculated energies with p*K*_a_ for 1,4-quinones

**Fig. 3:**
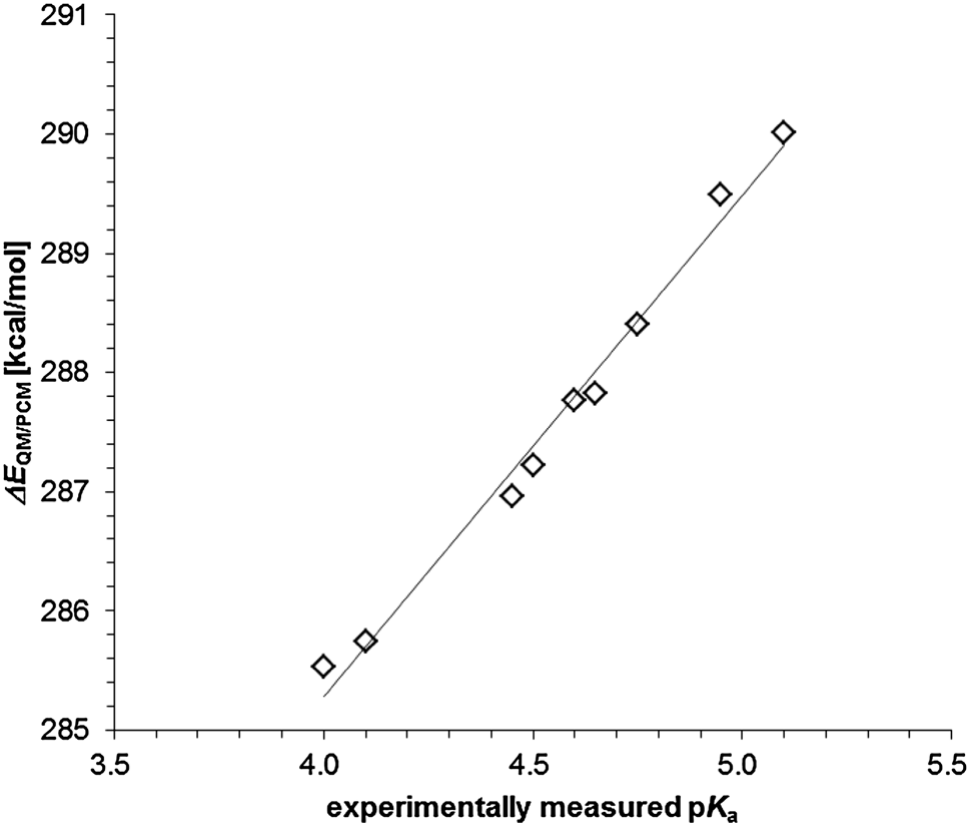
Correlation between experimentally measured p*K*_a_(Q^·−^/QH^·^) and calculated Δ*E*_QM/PCM_ (coefficient of determination *R* ^2^ = 0.99). Δ*E*_QM/PCM_ can be calculated using a quantum chemical approach with the PCM method. The *solid line* was drawn according to Eq. 4

The calculated Δ*E*_QM/PCM_ for deprotonation of QH^·^ to Q^·−^ for nine 1,4-quinones were highly associated with the experimentally measured p*K*_a_(Q^·−^/QH^·^) ranging from 4.0 to 5.1 (summarized in Swallow 1982), which was best fitted in the following equations (Fig. 3):

4 $$p{K_{\text{a}}}({{\text{Q}}^{ \cdot - }}/{\text{Q}}{{\text{H}}^ \cdot }){\text{ }}=\frac{1}{{4.20}}\left( {\Delta {E_{{\text{QM}}/{\text{PCM}}}} - 268.48\left[ {{\text{kcal}}/{\text{mol}}} \right]} \right).$$

The term of −268.48 kcal/mol corresponds to the proton solvation energy, which typically ranges from −252.6 to −271.7 kcal/mol [see Schmidt am Busch and Knapp (2004) and references therein]. Using Eq. 4, the calculated p*K*_a_(Q^·−^/QH^·^) for nine 1,4-quinones are listed in Table 1, which confirms that Eq. 4 can reproduce the experimentally measured p*K*_a_(Q^·−^/QH^·^). It should be noted that the accuracy of the experimentally measured p*K*_a_ values is generally considered to be within 0.2 units (Swallow 1982).

**Table 1:** Calculated p*K*_a_(Q^·−^/QH^·^) for the first protonation process Q^·−^ to QH^·^ in water, using Eq. 4

	p*K*_a_(Q^·−^/QH^·^)		
	Measured (in water)	Calculated (in water)	(Lower)
Benzoquinone	4^a,b,c^	4.06	n.d
Methylbenzoquinone	4.45^b^	4.40	(4.24)
2,3-Dimethylbenzoquinone	4.65^b^	4.60	n.d
2,5-Dimethylbenzoquinone	4.6^b,d,e^	4.59	n.d
2,6-Dimethylbenzoquinone	4.75^b^	4.74	(4.28)
Trimethylbenzoquinone	4.95^b^	5.00	(4.73)
Duroquinone	5.1^b,d,e^	5.13	n.d
1,4-Naphthoquinone	4.1^b,d,e^	4.11	n.d
2-Methyl-1,4-naphthoquinone	4.5^b,e^	4.46	(4.15)
Ubiquinone	n.d	5.31 at O4	(5.30 at O1)
Menaquinone/phylloquinone	n.d	4.92 at O4	(4.52 at O1)
Plastoquinone	n.d	5.11 at O4	(5.01 at O1)
Rhodoquinone	n.d	5.78 at O4	(4.23 at O1)

Lower p*K*_a_(QH^·^/Q^·−^) values, if available (i.e., asymmetrically shaped quinones), are listed in the bracket. Since protonation of Q^·−^ to QH^·^ occurs predominantly at one of the two O sites, O1 and O4, with a higher p*K*_a_(Q^·−^/QH^·^), the higher p*K*_a_(Q^·−^/QH^·^) can be considered to be experimentally measureable. Experimentally measured p*K*_a_(QH^·^/Q^·−^) are summarized in Swallow (1982). The error for nine quinones between experimentally measured and calculated p*K*_a_(Q^·−^/QH^·^) was 0.04 in p*K*_a_ unit. See Figs. 1 and 2 for the location of the O1 and O4 sites. n.d. = not determined

^a^Adams and Michael (1967)

^b^Patel and Willson (1973)

^c^Steenken and O’Neill (1977)

^d^Rao and Hayon (1973)

^e^Willson (1971)

Symmetrically shaped quinones (e.g., benzoquinone and 2,3-dimethylbenzoquinone) had a single p*K*_a_ value, whereas asymmetrically shaped quinones (e.g., methylbenzoquinone and 2,6-dimethylbenzoquinone) had two distinguishable p*K*_a_ values (Table 1). The difference in p*K*_a_ values is caused by the difference in the chemical environment of the two O sites (O1 and O4). For example, the calculated p*K*_a_(Q^·−^/QH^·^) of the O4 site in 2,6-dimethylbenzoquinone was larger than the calculated p*K*_a_(Q^·−^/QH^·^) of the O1 site by 0.46 (Table 1), because the protonation at O1 (i.e., the formation of –OH at O1) increases the steric hindrance with the two methyl groups, which results in a decrease in p*K*_a_ at O1. Since protonation of Q^·−^ to QH^·^ occurs predominantly at one of the two O sites with a higher p*K*_a_(Q^·−^/QH^·^), the higher p*K*_a_(Q^·−^/QH^·^) can be considered to be relevant for asymmetrically shaped quinones and are used to obtain Eq. 4.

**Fig. 4:**
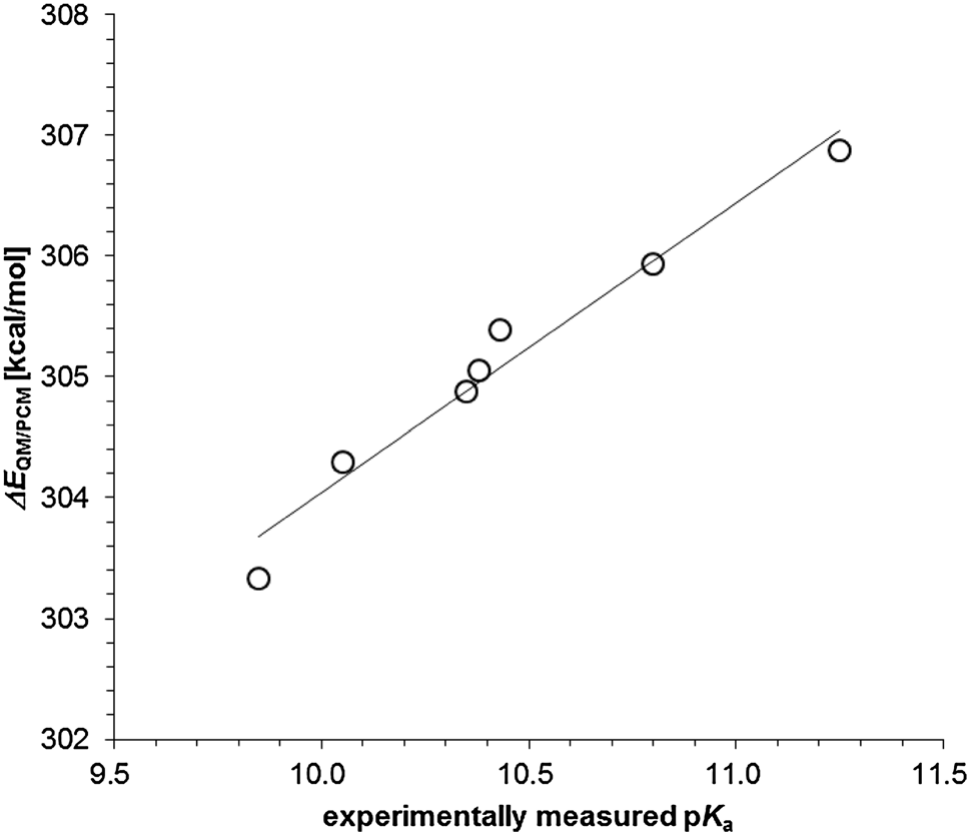
Correlation between experimentally measured p*K*_a_(QH^−^/QH_2_) and calculated Δ*E*_QM/PCM_ (coefficient of determination *R* ^2^ = 0.96). Δ*E*_QM/PCM_ can be calculated using a quantum chemical approach with the PCM method. The *solid line* is drawn according to Eq. 5

The calculated Δ*E*_QM/PCM_ for deprotonation of QH_2_ to QH^−^ for nine 1,4-quinones were also highly correlated with the experimentally measured p*K*_a_(QH^−^/QH_2_) ranging from 9.85 to 11.25 (Bishop and Tong 1965), which was best fitted in the following equations (Fig. 4):

5 $$p{K_{\text{a}}}({\text{Q}}{{\text{H}}^ - }/{\text{Q}}{{\text{H}}_2})=\frac{1}{{2.40}}\left( {\Delta {E_{{\text{QM}}/{\text{PCM}}}} - 280.00\left[ {{\text{kcal}}/{\text{mol}}} \right]} \right).$$

The p*K*_a_(QH^−^/QH_2_) calculated for nine quinones using Eq. 5 are listed in Table 2, which confirms that Eq. 5 can reproduce the experimentally measured p*K*_a_(QH^−^/QH_2_) when Δ*E*_QM/PCM_ can be calculated appropriately.

**Table 2:** Calculated p*K*_a_(QH^−^/QH_2_) for the second protonation process QH^−^ to QH_2_ in water, using Eq. 5

	p*K*_a_(QH^−^/QH_2_)	
	Measured (in water)	Calculated (in water)
Benzoquinone	9.85^a^	9.70
Methylbenzoquinone	10.05^a^	10.10
2,3-Dimethylbenzoquinone	10.43^a^	10.56
2,5-Dimethylbenzoquinone	10.38^a^	10.42
2,6-Dimethylbenzoquinone	10.35^a^	10.35
Trimethylbenzoquinone	10.8^a^	10.79
Duroquinone	11.25^a^	11.18
Ubiquinone	n.d	10.86
Menaquinone/phylloquinone	n.d	9.16
Plastoquinone	n.d	10.74
Rhodoquinone	n.d	9.81

The error for seven quinones between experimentally measured and calculated p*K*_a_(Q^·−^/QH^·^) was 0.09 in p*K*_a_ unit. *n.d*. not determined

^a^Bishop and Tong (1965)

### p*K*_a_(Q^·−^/QH^·^) and p*K*_a_(QH^−^/QH_2_) for ubiquinone, menaquinone, phylloquinone, plastoquinone, and rhodoquinone in aqueous solution

Using Eqs. 4 and 5, we also calculated p*K*_a_(Q^·−^/QH^·^) and p*K*_a_(QH^−^/QH_2_) for ubiquinone, menaquinone, phylloquinone, and plastoquinone in aqueous solution, respectively. The calculated p*K*_a_(Q^·−^/QH^·^) were 5.31 for ubiquinone, 4.92 for menaquinone/phylloquinone, and 5.11 for plastoquinone in aqueous solution (Table 1). p*K*_a_(Q^·−^/QH^·^) did not significantly differ between O1 and O4 in plastoquinone and ubiquinone (Table 1). This result suggests that which of the two O sites, O1 and O4, serves as the initial (i.e., Q^·−^ to QH^·^) and second (i.e., QH^−^ to QH_2_) protonation sites of Q_B_ in PbRC and PSII, is predominantly determined by the protein environments.

The calculated p*K*_a_(Q^·−^/QH^·^) for ubiquinone was closer to the value of 4.9 roughly estimated for aqueous solution (Swallow 1982) than the value of 6.5 measured in methanol (Land and Swallow 1970; Swallow 1982). Because methanol (dielectric constant = 33) is less polar than water (dielectric constant = 80), the negatively charged Q^·−^ state is less stable in methanol than in water; this could explain the high p*K*_a_(Q^·−^/QH^·^) in methanol with respect to water.

In theoretical studies by Cape et al., the calculated p*K*_a_(Q^·−^/QH^·^) of 5.09 for rhodoquinone was lower than their calculated p*K*_a_(Q^·−^/QH^·^) of 5.35 for ubiquinone (Cape et al. 2006). In contrast, in the present study, the calculated p*K*_a_(Q^·−^/QH^·^) of rhodoquinone was significantly high, 5.78 (Table 1). It should be noted that p*K*_a_(Q^·−^/QH^·^) of rhodoquinone is assumed to be higher than p*K*_a_(Q^·−^/QH^·^) of ubiquinone (e.g., Graige et al. 1999), which is consistent with the present result.

The calculated p*K*_a_(QH^−^/QH_2_) of 10.86 for ubiquinone (Table 2) is very close to p*K*_a_(QH^−^/QH_2_) of 10.8 measured for trimethylbenzoquinone (Bishop and Tong 1965). Zhu and Gunner considered p*K*_a_(QH^−^/QH_2_) for trimethylbenzoquinone being more relevant to the p*K*_a_(QH^−^/QH_2_) for ubiquinone in aqueous solution (Zhu and Gunner 2005) than p*K*_a_(QH^−^/QH_2_) = 13.3 in 80% ethanol (Morrison et al. 1982). The present study supports their conclusion. The calculated p*K*_a_(QH^−^/QH_2_) of 10.86 for ubiquinone is also close to the calculated p*K*_a_(QH^−^/QH_2_) of 10.74 for plastoquinone (Table 2), which might be associated with the similarity in the Q_B_ protonation events for PbRC and PSII (Robinson and Crofts 1984; Rutherford et al. 1984; Okamura et al. 2000; Wraight 2004; Ishikita and Knapp 2005).

### Influence of the 2-methoxy group orientation in p*K*_a_(Q^·−^/QH^·^) for ubiquinone

It was proposed that difference in the 2-methoxy orientation of ubiquinone (Fig. 1b) was responsible for (i) the *E*_m_ difference of more than 160 mV between Q_A_ and Q_B_ in PbRC (Taguchi et al. 2013) or (ii) the quantum chemically obtained electron affinity difference of more than 170 meV between Q_A_ and Q_B_ in PbRC (de Almeida et al. 2014). On the other hand, FTIR studies suggested that the Q_A_ binding to PbRC (i.e., interaction between Q_A_ and the PbRC protein environment) was affected by the H-bond interaction with the protein environment but not by the methoxy orientation (Remy et al. 2003). In the present study, using the crystal structure (PDB: 3I4D; Fig. 1b) whose different 2-methoxy orientations between Q_A_ and Q_B_ were highlighted in ref. (de Almeida et al. 2014), p*K*_a_(Q^·−^/QH^·^) were calculated. The calculated p*K*_a_(Q^·−^/QH^·^) were the same, 5.30 at the O4 site in the Q_A_ conformation and 5.31 at the O4 site in the Q_B_ conformation in water (Table S1), irrespective of the different 2-methoxy orientations in the quantum chemically optimized quinone structures (SI Dataset 2 for atomic coordinates). In the protonated QH^·^ state, the energetically lowest conformations showed that the –OH group was oriented toward the 2-methoxy O atom (–O–H⋯O_methoxy_ angle = 116° and –O⋯O_methoxy_ distance = 2.1 Å in both Q_A_ and Q_B_ conformations). When the –OH group was oriented away from the 2-methoxy O atom, the calculated p*K*_a_(Q^·−^/QH^·^) were lowered by ~0.6 to 1 p*K*_a_ unit from the p*K*_a_(Q^·−^/QH^·^) value of the energetically lowest conformation (Table S1). In the quantum chemically optimized Q_A_ and Q_B_ conformations, the 2-methoxy group was out of the quinone ring, as identified in the crystal structure (PDB: 3I4D). These results suggest that p*K*_a_(Q^·−^/QH^·^) is more affected by the difference in the –OH orientation than the difference in the 2-methoxy orientation. The influence of the 2-methoxy orientation might be more pronounced when calculated in vacuum (i.e., in the absence of the protein environment), where H-bond partners of ubiquinone (e.g., bulk water molecules and protein environments) are absent. In vacuum, alteration in the molecular configuration is the only way to alter the stability of the Q^·−^ and QH^·^ states. It should be noted that the influence of –OH orientation can be ignored when considering *E*_m_(Q/Q^·−^), because –OH is absent in both the Q and Q^·−^ states.

### Evolutionary transition of quinones in photosynthetic reaction centers

Among all of the 1,4-quinones investigated here, plastoquinone and ubiquinone employed at Q_B_ in PbRC and PSII have the second and third largest p*K*_a_(Q^·−^/QH^·^) (Table 1). This might be a reason why nature uses these quinones exclusively as the terminal electron acceptor Q_B_ in PbRC and PSII. The large p*K*_a_(Q^·−^/QH^·^) would result in a larger population of QH^·^. This will then enable it to accept the second electron, leading to the fully protonated Q_B_H_2_ state. Thus, the large p*K*_a_(Q^·−^/QH^·^) is advantageous in fixing the two transferred electrons using protonation in the form of Q_B_H_2_. Remarkably, Q_A_ in PbRC from *B. viridis* and A_1A_ and A_1B_ in PSI, which serve as electron donors (to Q_B_ and the Fe_4_S_4_ cluster F_X_, respectively, Fig. 1) without altering the protonation states, are menaquinone/phylloquinone with lower p*K*_a_(Q^·−^/QH^·^) = 4.9 (Table 1).

Menaquinone is assumed to represent the ancestral type of quinones in bioenergetics systems, whereas ubiquinone and plastoquinone are assumed to represent “more recent” quinones (Schoepp-Cothenet et al. 2009). Intriguingly, *E*_m_(Q/Q^·−^) of the “more recent” quinones are higher [by ~100 mV (Prince et al. 1983)] than *E*_m_(Q/Q^·−^) of the “ancestral” quinone. Reduced menaquinone becomes rapidly oxidized in the presence of O_2_, whereas ubiquinone and plastoquinone are less sensitive toward oxygen, minimizing loss of the unproductive reduced power (Schoepp-Cothenet et al. 2009); it seems plausible that the difference in *E*_m_(Q/Q^·−^) is associated with rising levels of dioxygen 2.5 billion years ago. In most PbRC, the tightly bound cofactor at the Q_A_ site can be either menaquinone or ubiquinone, whereas the cofactor at the terminal electron acceptor Q_B_ site is ubiquinone [except for, e.g., PbRC from *Halorhodospira halophile*, which has menaquinone at both the Q_A_ and Q_B_ site (Schoepp-Cothenet et al. 2009)]. The higher *E*_m_(Q/Q^·−^) of ubiquinone at the Q_B_ site would contribute to the driving force of the electron transfer from Q_A_ to Q_B_ when menaquinone is the cofactor at the Q_A_ site (e.g., *B. viridis*). The present results show that the “more recent” quinones, ubiquinone and plastoquinone, have larger p*K*_a_(Q^·−^/QH^·^) and p*K*_a_(QH^−^/QH_2_) values than the “ancestral” quinone, menaquinone/phylloquinone (Tables 1, 2), which is also consistent with a role of Q_B_, fixation of two electrons and two protons in the form of Q_B_H_2_.

In photosynthetic reaction centers of the green non-sulfur bacteria (e.g., *Chloroflexus aurantiacus*), which is assumed to have evolved first as the type-II reaction center (Gupta 2003), menaquinone occupies at both the Q_A_ and Q_B_ sites (Hale et al. 1983). One of the simplest ways to generate the driving force for electron transfer between the identical menaquinone cofactors would be to alter the solvent accessibility between the Q_A_ and Q_B_ sites. As the quinone-binding site is more exposed to the bulk water region, it can stabilize the Q^·−^ state with respect to Q state, which results in an increase in *E*_m_(Q), i.e., the case for *E*_m_(Q_B_), even if the amino acid sequences near the Q_A_ and Q_B_ binding sites were similar. In addition, the exposure of the quinone-binding site makes (P)bRC easier to hire external quinones. The effort to make the solvent accessibility of the quinone-binding sites different between the two quinone-binding sites in the homodimeric type-I reaction center would lead to formation of the heterodimeric type-II reaction center with *E*_m_(Q_B_) > *E*_m_(Q_A_) and might also explain why the cofactor can be ubiquinone at the Q_B_ site in most PbRC.

## Conclusions

Experimentally measured p*K*_a_(Q^·−^/QH^·^) (Swallow 1982) and p*K*_a_(QH^−^/QH_2_) (Bishop and Tong 1965) values of nine 1,4-quinones in aqueous solution were highly correlated with the quantum chemically calculated energy differences (Δ*E*_QM/PCM_) between the protonated and deprotonated states (Figs. 3, 4). They can be best fitted to Eqs. 4 and 5, respectively. The calculated p*K*_a_(Q^·−^/QH^·^) values were 5.31 for ubiquinone, 4.92 for menaquinone/phylloquinone, 5.11 for plastoquinone, and 5.78 for rhodoquinone in aqueous solution (Table 1). p*K*_a_(Q^·−^/QH^·^) for plastoquinone and ubiquinone in aqueous solution are the largest among all of the 1,4-quinones but rhodoquinone listed in Table 1, partially explaining why nature employs these two 1,4-quinones specifically as the terminal electron acceptors Q_B_ in PbRC and PSII.

In PbRC and PSII, the initial protonation of Q^·−^ to QH^·^ predominantly occurs at the distal carbonyl site O_dist_ of Q_B_ (Fig. 1) (Okamura et al. 2000; Wraight 2004; Saito et al. 2013). The p*K*_a_(Q^·−^/QH^·^) at the O1 and O4 sites did not differ significantly for each quinone (Table 1), suggesting that the protein environments predominantly determine the initial protonation O site of Q_B_.

The p*K*_a_(QH^−^/QH_2_) values were calculated to be 10.86 for ubiquinone, 9.16 for menaquinone/phylloquinone, 10.74 for plastoquinone, and 9.81 for rhodoquinone in aqueous solution using Eq. 5 (Table 2). The calculated p*K*_a_(QH^−^/QH_2_) for ubiquinone was closer to the experimentally measured value of 10.8 for trimethylbenzoquinone (Bishop and Tong 1965) than the experimentally measured p*K*_a_(QH^−^/QH_2_) of 13.3 for ubiquinone in 80% ethanol (Morrison et al. 1982), as already pointed out (Zhu and Gunner 2005).

The p*K*_a_(Q^·−^/QH^·^) and p*K*_a_(QH^−^/QH_2_) for ubiquinone, menaquinone/phylloquinone, and plastoquinone in aqueous solution, which were determined in the present study, will help understand the mechanisms of quinone-mediated reactions in photosynthetic reaction centers, such as formation of Q_B_H_2_ in PbRC and PSII (Robinson and Crofts 1984; Rutherford et al. 1984; Okamura et al. 2000; Wraight 2004; Saito et al. 2013), formation of Q_A_H_2_ in PSII (van Mieghem et al. 1995; Noguchi 2002), and evolutionary relationship between the type-I and type-II photosynthetic reaction centers (Gupta 2003; Rutherford and Faller 2003; Schoepp-Cothenet et al. 2009).

## Electronic supplementary material

Below is the link to the electronic supplementary material.

Supplementary material 1 (DOC 67 KB)

Supplementary material 1 (PDB 52 KB)

Supplementary material 1 (PDB 49 KB)
