# Supplementary material for: pKa of ubiquinone, menaquinone, phylloquinone, plastoquinone, and rhodoquinone in aqueous solution
Source: Photosynth Res. 2017 Apr 12;133(1):297–304. doi: 10.1007/s11120-017-0382-y (PMC5500672; doi:10.1007/s11120-017-0382-y)
Supplement: Supplementary file 1 — Supplementary material 1 (DOC 67 KB) [file 11120_2017_382_MOESM1_ESM.doc]

**Table S1.** Calculated p*K*_a_(Q^●–^/QH^●^) for ubiquinone, using the using the crystal structure (PDB: 3I4D). The energetically lowest conformations are in bold (UBIQUINONEA-4 for Q_A_ and UBIQUINONEB-4 for Q_B_). –OH orientation; toward = –OH group is oriented toward the 2-methoxy O atom; away = –OH group is oriented away the 2-methoxy O atom.

| **Original structure** | **In Dataset 2** |  | |  |  |
| --- | --- | --- | --- | --- | --- |
| **(PDB: 3I4D)** | **name** | **model**  **(Q^●–^)** | **model**  **(QH^●^)** | **–OH**  **orientation** | **p*K*_a_** |
| Q_A_ | UBIQUINONEA-1 | 1 | 2 | away | 4.24 |
| Q_A_ | UBIQUINONEA-2 | 3 | 4 | toward | 5.06 |
| Q_A_ | UBIQUINONEA-3 | 5 | 6 | away | 4.68 |
| **Q_A_** | **UBIQUINONEA-4** | **7** | **8** | **toward** | **5.30** |
| Q_B_ | UBIQUINONEB-1 | 9 | 10 | away | 4.30 |
| Q_B_ | UBIQUINONEB-2 | 11 | 12 | toward | 5.27 |
| Q_B_ | UBIQUINONEB-3 | 13 | 14 | away | 4.69 |
| **Q_B_** | **UBIQUINONEB-4** | **15** | **16** | **toward** | **5.31** |
